# Refuting Six Misconceptions about Romantic Love

**DOI:** 10.3390/bs14050383

**Published:** 2024-05-02

**Authors:** Sandra J. E. Langeslag

**Affiliations:** Department of Psychological Sciences, University of Missouri—St. Louis, St. Louis, MO 63121, USA; langeslags@umsl.edu

**Keywords:** love, close relationships, misconceptions, myths, emotions, brain, neuroscience

## Abstract

Scientific research on romantic love has been relatively sparse but is becoming more prevalent, as it should. Unfortunately, several misconceptions about romantic love are becoming entrenched in the popular media and/or the scientific community, which hampers progress. Therefore, I refute six misconceptions about romantic love in this article. I explain why (1) romantic love is not necessarily dyadic, social, or interpersonal, (2) love is not an emotion, (3) romantic love does not just have positive effects, (4) romantic love is not uncontrollable, (5) there is no dedicated love brain region, neurotransmitter, or hormone, and (6) pharmacological manipulation of romantic love is not near. To increase progress in our scientific understanding of romantic love, I recommend that we study the intrapersonal aspects of romantic love including the intensity of love, that we focus our research questions and designs using a component process model of romantic love, and that we distinguish hypotheses and suggestions from empirical findings when citing previous work.

## 1. Introduction

There is a growing interest in the science of romantic love, as evident from the increased number of publications on this topic, the organization of scientific conferences devoted to research on love, and the publication of special journal issues like this one. This growing interest is exciting and warranted for two reasons. First, love pertains to virtually everyone. For example, more than 80% of adolescents in a study in the US reported to have been involved in at least one romantic relationship by the age of 18 [1] and love has been observed in almost all cultures that have been studied [2]. Second, when people fall in love, it greatly impacts their lives. People are sometimes even willing to change their friends, job, country, or religion to be with their beloved [3]. It is important that we conduct thorough scientific research on romantic love and that we disseminate the results broadly. Unfortunately, there are several misconceptions about romantic love that are permeating popular media, the scientific community, or both. Some of these misconceptions stem from lay people’s and scientists’ assumptions about romantic love. Other misconceptions stem from hypotheses or interpretations put forth in scientific articles being cited in other articles as empirical evidence. Collectively, these misconceptions are hampering the progress of the scientific understanding of romantic love. In this article, I refute six of those misconceptions and provide recommendations for research.

The misconceptions refuted in this article concern romantic love, which is love for a significant other (as opposed to love for family members or friends, for example) and can be experienced regardless of whether someone is in a romantic relationship with the beloved. Scientists have proposed several taxonomies of love, each with various numbers and different types of love, e.g., [4,5,6,7]. Because the word “love” does not have a plural form, I use the term “love feelings” to refer to a collection of different love types (note that the term “feeling” does not necessarily refer to an emotion [8]). In my work, I have distinguished between the following types of romantic love: infatuation (or passionate love), attachment (or companionate love), and sexual desire (cf. [5]). Infatuation is the overwhelming, amorous feeling for one individual that is typically most intense during the early stage of love (i.e., when individuals are not (yet) in a relationship with their beloved or are in a new relationship). Attachment, on the other hand, is the comforting feeling of emotional bonding with another individual that takes some time to develop, often in the context of a romantic relationship [5,6,9]. Sexual desire is the craving for sexual gratification [5]. Even though some of the arguments below may depend on certain definitions or taxonomies of love, those arguments are never the sole argument used to refute a misconception. Therefore, the overall refutation of each misconception does not hinge on specific definitions or taxonomies of love.

## 2. Romantic Love Is Not Necessarily Dyadic, Social, or Interpersonal

The first misconception is that romantic love is dyadic, social, or interpersonal, and that it only exists within romantic relationships. For example, one anonymous reviewer of one of my manuscripts commented that “It’s odd that ~1/6 of the sample who were purportedly “in love” were not in a relationship with the target of their love.” While romantic love has obvious interpersonal aspects (i.e., people are in love with another person and romantic relationships involve more than one person by definition), romantic love is not *necessarily* dyadic, social, or interpersonal, and can be experienced outside of the context of a romantic relationship.

In contrast to what the reviewer seemed to think, it does not take two to love. For example, people may develop love feelings for someone before they become involved in a romantic relationship with that person [9], or may still experience love feelings after a romantic relationship has ended [10]. People may experience love feelings that are not reciprocated and may love someone they have never been and will never be in a romantic relationship with. People can even experience love feelings for someone they have never interacted with. For example, people can experience love at first sight [11] and some people develop one-sided, parasocial attachment to celebrities [12]. Interestingly, people even report experiencing parasocial attachment to fictional characters in movies, TV shows, video games, and books [13,14]. Finally, people may experience sexual arousal in the absence of (thoughts of) another individual, such as during masturbation, which suggests that sexual desire, which has been considered a type of love by some researchers [5], may not require a target at all.

So, romantic love is not necessarily dyadic, social, or interpersonal, and does not only exist within romantic relationships. Therefore, we need to broaden our approach and study the intrapersonal aspects of romantic love as well. This does not require the study of dyads who are in a relationship with each other, but can be achieved by studying individuals who are experiencing love feelings, regardless of their relationship status. This also means that besides fitting into the realm of social psychology/neuroscience, research on romantic love fits into the realm of affective and cognitive (neuro)science.

Due to the social emphasis of previous research on romantic love, researchers have often focused on relationship satisfaction rather than intrapersonal love feelings. It is important to note, though, that romantic love is not the same as relationship satisfaction. People can be satisfied with a relationship if it fulfils some need (e.g., money, housekeeping, sex, protection, child care, status, personal growth), rather than experiencing love for their partner [15]. And, in abusive relationships, conversely, it is possible that the victim is in love with their abuser while being unsatisfied with the relationship. In a sample of married individuals, the correlation between relationship satisfaction and attachment level was high but nowhere near perfect (.68), and the correlation between relationship satisfaction and infatuation level was only .18 [16]. And, in a meta-analysis of 25 questionnaire studies, the correlations between relationship satisfaction and the intensity of different types of love (i.e., infatuation, attachment, eros, and mania) ranged between −.02 and .56 [17]. These findings confirm that even though relationship satisfaction and intensity of love feelings may be related, they are distinct concepts.

Because romantic love is distinct from relationship satisfaction, relationship satisfaction—while interesting in its own right—should not be used as a proxy for love intensity in research. Relationship satisfaction can be assessed using items such as “How happy/satisfied are you in your relationship with___?” or using questionnaires such as the Couples Satisfaction Index (CSI-32) [18] or the Revised Dyadic Adjustment Scale (RDAS) [19]. Intensity of love feelings, on the other hand, can be assessed using items such as “How in love with/infatuated with/attached to___are you?” or questionnaires such as Infatuation and Attachment Scales [9].

## 3. Love Is Not an Emotion

The second misconception is that love is an emotion (similar to fear, anger, sadness, surprise, disgust, and joy, for example). Lay people typically consider love to be an emotion [20,21] and so do some scientists [22,23]. Although it depends on how emotions are defined, there are several reasons to assume that love is not an emotion.

As mentioned above, although scientists do not agree on how many and which types of love exist, they do agree that there are multiple types of love [4,5,6,7]. There being multiple types of love is one reason to assume that love *as a whole* is not an emotion [24]. There are also reasons to assume that the different types of love themselves are not emotions either. First, love elicits various emotions depending on the situation. When infatuation is requited, for example, it may elicit the emotion joy [25], yet it may elicit the emotion sadness when it is unrequited [26]. Note that this is different from how emotions can elicit meta-emotions, which are emotions about one’s own emotions [27]. An example of a meta-emotion is when someone is anxious about an upcoming presentation and then feels shame about being anxious. The emotions elicited as a result of love, in contrast, are not *about* being in love. For example, someone who is in love may feel jealous when their beloved attends to others, but they are not jealous about being in love. Second, it has been shown that distraction after a romantic breakup decreased negative affect but not the intensity of love, and that negative reappraisal of an ex-partner decreased love intensity yet increased negative affect [10,28]. These observed dissociations indicate that love regulation and emotion regulation are conceptually distinct. That is, love regulation targets love feelings (such as infatuation and attachment), whereas emotion regulation targets emotions (such as fear, anger, sadness, surprise, disgust, and joy) [10]. Third, another reason why love feelings are not emotions is that love feelings can be very long lasting, whereas emotions are usually shorter lasting. Research has shown that emotions typically last for a half hour up to several days. The longest lasting emotion was sadness, with a median duration until a first return to baseline of 48 h (i.e., 2 days) and a median duration until permanent return to baseline of 120 h (i.e., 5 days) [29]. In contrast, it is not uncommon for infatuation to last for weeks or months and for attachment to last for years or decades [9,30].

Rather than an emotion, scientists have categorized love as an attitude [31], a script [32], or a motivation or drive (similar to craving, hunger, and thirst) [24,33,34,35]. Although refuting the misconception that love is an emotion may just seem a quibble about semantics, it does suggest that we should not assume that any knowledge we have about emotions generalizes to romantic love.

## 4. Romantic Love Does Not Just Have Positive Effects

The third misconception is that romantic love has mainly positive effects, which has contributed to the relative paucity of scientific interest in romantic love (e.g., in terms of the number of publications, journals, scientific societies, and grant mechanisms). Of course, love has a plethora of positive effects on people and society. Infatuation, for example, elicits positive emotions such as euphoria [25], and romantic relationships enhance happiness and life satisfaction [36]. It is often overlooked, however, that love has many negative effects on people and society as well.

First, love can elicit several negative emotions. For example, infatuation is stressful [37], love can be accompanied by jealousy [38], the death of a romantic partner may elicit intense grief [39], and unreciprocated love and romantic breakups elicit sadness and shame [26]. Second, love may reduce general well-being. For example, romantic breakups are a main risk factor for major depressive disorder in adolescents [40] and dysfunctional romantic relationships and romantic breakups are associated with decreased happiness and life satisfaction [41,42]. Third, people who are in love may be distracted from their duties (e.g., work or homework) because they think about their beloved all the time [43,44]. Even though this may not bother the infatuated individual, it may result in a loss of productivity. Fourth, love plays a role in several mental disorders, including sexual dysfunctions, paraphilic disorders, and erotomanic and jealous delusional disorders [45], as well as in suicide behavior [46]. Finally, love is associated with various forms of criminal behavior including stalking [47], domestic violence [48,49], and homicide [50].

It may be clear that love has both positive and negative effects. Taken together, the negative effects of love cause substantial individual, social, and economic burden, and underscore the great need to study romantic love. It is my hope that thorough research on romantic love can both increase the positive effects of love and decrease its negative effects on individuals and society.

## 5. Romantic Love Is Not Uncontrollable

The fourth misconception is that love should not and/or cannot be controlled. An anonymous reviewer for one of my grant proposals on love regulation asked: “Do we want to be able to control our love feelings?”

Indeed, it is not uncommon for people to think that love should not be controlled because it is a natural process [28]. Nevertheless, there are many situations in which it might be beneficial to regulate love feelings. For example, love feelings may be stronger than desired, such as when people are still in love with their ex-partner after a breakup, when the love is forbidden, and when people are in love with someone who treats them poorly. In situations like those, people may want to decrease how in love they are. Successful down-regulation of love feelings could ameliorate heartbreak. It could also help people to stop pursuing an inappropriate partner or to put an end to a dysfunctional (e.g., abusive) relationship. At other times, love feelings may be weaker than desired, such as when they decline in long-term relationships [9,30]. Successful up-regulation of love may stabilize long-term relationships.

In addition, some people think that love regulation is difficult or even impossible [25,28]. People think that other people are better at love regulation than they are, that who they are in love with is less controllable than love intensity, and that infatuation is even less controllable than attachment and sexual desire [10,28,51,52]. Nevertheless, a series of studies have shown that people *can* control different types of love using behavioral and cognitive strategies. Specifically, looking at pictures of the beloved increases infatuation and attachment [16], positive reappraisal of the beloved, the relationship, and/or the future increases attachment [28,53], and sexual imagery increases sexual desire and infatuation [53]. Conversely, negative reappraisal of the beloved, the relationship, and/or the future decreases infatuation and attachment [10,28].

In short, misconceptions that love regulation is undesirable and/or difficult exist. Given that love regulation is possible and can be adaptive, these misconceptions have negative consequences for well-being. Therefore, lay people need to be educated about the feasibility and benefits of love regulation, and scientists should not let their own misconceptions keep them and others from studying this important topic.

## 6. There Is No Dedicated Love Brain Region, Love Neurotransmitter, or Love Hormone

The fifth misconception is that there is a dedicated love brain region, love neurotransmitter, and/or love hormone. This misconception is more prevalent in lay people than in scientists (the #1 question that I am asked in interviews for popular media is which brain region is involved in love) and reflects a lack of knowledge of how the brain and body work more than a misconception about love specifically.

Generally, there is no one-to-one mapping between anatomy and function. Each brain region, neurotransmitter, and hormone has multiple functions, and each function requires multiple brain regions, neurotransmitters, and/or hormones. Love affects behaviors, feelings, cognition, and physiology in many different ways [25]. Each of these ‘components’ of romantic love depends on a different network of brain regions, and multiple neurotransmitters and/or hormones. Take, for example, the enhanced memory for information that is related to the beloved, which is related to the arousal that this information elicits [54]. We know that enhanced memory for arousing information depends on the amygdala and hippocampus, the neurotransmitter noradrenaline, and the hormones adrenaline and cortisol [55,56]. Therefore, it can be expected that those brain regions, neurotransmitters, and hormones are involved in the memory bias for the beloved. And, take the increased skin conductance response to the beloved [57]. We know that skin conductance responses result from the release of the neurotransmitter acetylcholine in the sympathetic nervous system, which, in turn, is innervated by the paraventricular nucleus of the hypothalamus [58]. Therefore, that brain region and neurotransmitter likely play a role in the skin conductance response to the beloved. I hope that these two examples clarify how different brain networks, neurotransmitters, and hormones are involved in the different components of romantic love. This component process model reflects an approach that aims to understand romantic love as an emergent process that consists of numerous components, each with its own neurobiological basis.

Even though scientists typically understand that there is no dedicated love brain region, love neurotransmitter, or love hormone, they could improve the focus of their research questions and designs according to the component process model. Many neuroimaging studies on romantic love (including some of my own) use a passive viewing design, which makes it difficult to interpret the findings without resorting to reverse inference, which is the inference of mental states from neuroimaging data [59]. By having participants complete specific tasks during neuroimaging (e.g., a memory, attention, or regulation task) instead, the findings become more interpretable in terms of mental states, which will then allow us to learn that brain network/neurotransmitter/hormone X plays a role in component Y of romantic love.

## 7. Pharmacological Manipulation of Romantic Love Is Not Near

The final misconception is that our recent knowledge of the biological basis of romantic love will soon lead to a pharmacological route to control romantic love [60,61,62,63], which is something that people have pursued for ages. Even nowadays, people across the world wishfully use aphrodisiac substances and love philters [64]. However, although evidence-based pharmacological manipulation of love feelings may be feasible at some point in the future, several issues prevent the development of an effective and safe ‘love pill’ in the short term.

First, the scientific community has only just begun to unravel the psychopharmacology of love. We are only starting to learn which neurotransmitters and hormones might play a role in the different types of love. For example, several functional Magnetic Resonance Imaging (fMRI) studies have shown that brain regions such as the caudate, putamen, ventral tegmental area, insula, cingulate cortex, and inferior frontal gyrus are more active when people view pictures of their beloved than when they view pictures of other people [65,66,67,68,69,70,71,72]. Some of those regions (especially the caudate, putamen, and ventral tegmental area) are dopaminergic brain regions, and their activation in response to the beloved has been interpreted as evidence for the notion that romantic love is associated with increased levels of the neurotransmitter dopamine. However, it is important to note that fMRI studies cannot reveal whether dopamine is released in response to the beloved. To my knowledge, there is only one study that has actually measured dopamine levels when people view pictures of their beloved (compared to when they view pictures of friends), using positron emission tomography (PET). That study showed more dopamine release in response to the beloved pictures than the friend pictures in the medial orbitofrontal and prefrontal cortices, but surprisingly not in the more typical dopaminergic regions [73]. So, more research is needed on whether and where dopamine is released when people see their beloved. As another example, it has been hypothesized that romantic love is associated with reduced levels of serotonin because of its resemblance with obsessive–compulsive disorder [25]. In one study, however, women who were in love had higher blood serotonin levels than women who were not in love, and obsessive thinking about the beloved in women was associated with an increased serotonin level in serum [43]. So we cannot conclude at this time that romantic love is associated with reduced serotonin levels. In addition, we are still far from understanding any *causal* relationships between psychopharmacology and love feelings. For example, many studies compare people who are in love when they view stimuli that are related to their beloved with when they view other stimuli. It would be informative, but more difficult, to compare participants who are in love with participants who are not in love or even to compare people before and after they fall in love. Crucially, in order to develop a ‘love pill’ we would have to prove that alteration of neurotransmitter or hormone levels actually results in a change in the intensity of love.

Second, it would be challenging to design a drug that targets love feelings for one person specifically, which would be desirable in at least some situations. For example, someone who is married might want to decrease their love feelings for a crush without changing (or while increasing) their love for their spouse. Third, because the neurotransmitters and hormones involved in love have many different functions, any love drug that affects the levels of these neurotransmitters or hormones may have side effects that could be adverse. Finally, pharmacological manipulation of love feelings is associated with ethical issues such as who decides whether and when someone takes a love drug to increase or decrease their love feelings [60,61]. Compared to pharmacological manipulation of love feelings, behavioral and cognitive strategies to regulate love feelings like the ones mentioned before (i.e., looking at pictures of the beloved, positive or negative reappraisal of the beloved, the relationship, and/or the future, and sexual imagery) are associated with fewer physical and ethical risks and can be implemented right away.

In addition, I hope that the examples about dopamine and serotonin show that we need to be careful when citing previous work. That is, we should be specific when describing previous research findings (e.g., dopaminergic regions becoming more active vs. dopamine levels being elevated). Additionally, when citing researchers‘ suggestions and hypotheses (e.g., love being associated with high dopamine and low serotonin levels), we need to label them as such so that they are not taken as empirical evidence by the reader.

## 8. Conclusions

Science is making great strides in understanding romantic love. In this article, I have refuted six common misconceptions that exist in lay people and/or scientists, in the hopes that this will allow us to make even greater progress. Alongside the social science of romantic relationships, the cognitive and affective neuroscience of romantic love could mature into its own important field of research that focuses on the intrapersonal aspects of romantic love, including the intensity of love. Different types of love and the various emotions have some similarities, so we can be inspired by our knowledge of emotions, but because love is not an emotion we cannot assume that anything we know about emotions also applies to romantic love. We need to realize that love has multiple negative effects, which underscores the importance of research on this topic. And despite what people may think, it is possible and may be beneficial to control love feelings. Future research can explore what strategies are effective and adaptive in which situations. I also recommend focusing our research questions and designs using a component process model of romantic love. Finally, more work needs to be done to understand the (causal) role that various brain networks, neurotransmitters, and hormones play in romantic love. When citing previous work, we need to distinguish suggestions from empirical findings and recognize the limits of information that a certain research method provides.

To conclude, research on romantic love is extremely important because it pertains to almost everyone and because it affects people to a great extent, both good and bad. I recommend that we do not let our misconceptions guide what we study and that we cite previous work precisely, so that the science of romantic love can be an even more fruitful field of research that will benefit individuals and societies.

## Data Availability

No new data were created or analyzed in this study. Data sharing is not applicable to this article.
